# Severe heparin-induced thrombocytopenia: when the obvious is not obvious, a case report

**DOI:** 10.1186/1752-1947-1-13

**Published:** 2007-04-30

**Authors:** Graham M Cormack, Larry J Kaufman

**Affiliations:** 1 Department of Medicine, University of Hawaii, and St. Francis Medical Center, Honolulu, HI, USA

## Abstract

Thrombocytopenia commonly occurs in hospitalized patients, particularly critically ill patients. We present an exemplifying case of severe heparin-induced thrombocytopenia (HIT) in an effort to solidify its high priority in the differential diagnosis of thrombocytopenia. A 75-year-old female underwent cardiac surgery with intraaortic balloon pump (IABP) placement. A platelet count drop to 25 × 10(9)/L by the third postoperative day was attributed to the IABP, which was removed. Her thrombocytopenia remained refractory to multiple platelet transfusions over several days. Right hand cyanosis then developed, attributed to a right radial arterial catheter, which was removed. All toes and fingers then showed severe ischemic changes. Ten days after the initial platelet count drop, a critical care specialist new to the treating team suspected HIT. Heparin exposure was stopped and argatroban was initiated. A HIT antibody test was subsequently strongly positive. The patients thrombocytopenia gradually resolved. No additional thromboses occurred during a 27-day intensive care unit stay. This case underscores the need for vigilance in suspecting HIT in patients with thrombocytopenia and recent heparin exposure. To avoid catastrophic outcomes in such patients, heparin should be stopped and alternative anticoagulation should be initiated, at least until HIT is excluded.

## Background

Thrombocytopenia is a common finding in hospitalized patients, particularly critically ill patients, with readily plausible causes including disseminated intravascular coagulation, dilution from blood transfusions, continuous venovenous hemodialysis (CVVHD), liver disease with hypersplenism, and certain medications. Physicians should be vigilant in excluding the more dangerous causes of thrombocytopenia. For example, heparin-induced thrombocytopenia (HIT) warrants serious consideration because it is a potentially devastating, yet underdiagnosed, complication of one of the most commonly prescribed medications worldwide.

Approximately one trillion units of heparin are administered to 12 million patients per year in the United States [1]. HIT is caused by antibodies to a complex of heparin and platelet factor 4 that activate platelets, resulting in release of procoagulant microparticles, thrombocytopenia, excessive thrombin generation, and frequently thrombosis [2]. HIT occurs in approximately 0.5% of patients with occult exposure to heparin (e.g., catheter flushes), 0.1%–1% of patients treated with low-molecular-weight heparin [3], and 3%–5% of patients receiving unfractionated heparin [4]. Although these are small percentages, the ubiquitous use of heparin puts an extremely large number of patients at risk.

We present a case of severe HIT complicated by a highly hypercoagulable state, in which heparin exposure was inconspicuous and diagnosis was delayed. This case underscores the need for vigilance in suspecting HIT in any patient with thrombocytopenia and recent heparin exposure.

## Case report

A 75 year-old Hawaiian-Chinese female with a history of aortic stenosis, chronic renal insufficiency, and hypertension presented to her cardiologist with pitting edema of the bilateral lower extremities. On March 21^st^, 2005, a cardiac catheterization showed an ejection fraction of 15%, and severe aortic stenosis, aortic regurgitation, and mitral regurgitation. During catheterization, the venous and arterial sheaths were each flushed with approximately 250 units of heparin. Elective aortic valve replacement and mitral valve repair surgery with intraaortic balloon pump (IABP) placement was performed 10 days later. While on cardiopulmonary bypass, she received 32,000 units of heparin.

The patient's preoperative platelet count of 108 × 10^9^/L fell to 25 × 10^9^/L by the third postoperative day. She was transfused with 12 units of random donor platelets and received norepinephrine for blood pressure support. The decrease in platelets was attributed to the IABP, which subsequently was removed. The patient bled from the femoral insertion site and developed further hypotension. She underwent surgical repair of the femoral artery, during which she received 18 units of random donor platelets, 8 units of packed red blood cells, and 4 units of fresh frozen plasma. Her renal function deteriorated, necessitating CVVHD. A heparin-flushed dialysis catheter was placed, and the patient was exposed to additional heparin in the CVVHD tubing circuit.

**Figure 1:**
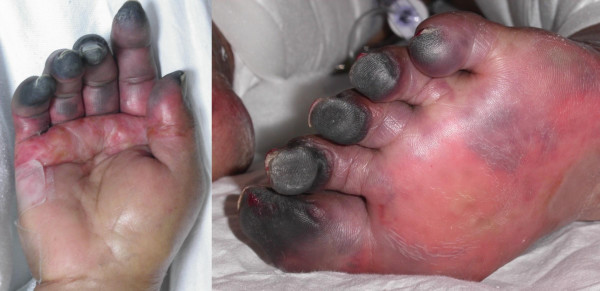
Gangrenous right hand and left foot as they appeared on hospital day #15.

Seven days postoperatively, the platelet count remained low (43 × 10^9^/L) despite a cumulative transfusion total of 48 units of random donor platelets. Differential diagnostic considerations by the patient's consultants included accelerated platelet removal from the circulation owing to CVVHD and sepsis-related disseminated intravascular coagulation. Two days later, right hand cyanosis was noted and attributed to the presence of a right radial arterial catheter, which was removed the next day without improvement. By then, all toes and fingers showed severe ischemic changes (Figure 1). Two days later, the platelet count reached its nadir (8 × 10^9^/L), resulting in more platelet transfusions.

A Critical Care specialist joined the multi-physician team the next day, and prompted by the ischemic physical findings, ordered a heparin-platelet factor 4 enzyme-linked immunosorbent assay (ELISA) which later proved strongly positive. The direct thrombin inhibitor argatroban was immediately begun at the recommended starting dose for patients without hepatic impairment (2 mcg/kg/min) and titrated downward over the next few days (lowest dose, 0.25 mcg/kg/min) because of activated partial thromboplastin times (aPTTs) of up to 200 seconds (baseline aPTT 35.1 seconds). Laboratory values suggested fairly normal liver function (aspartate aminotransferase 32 IU/L, alanine aminotransferase 17 IU/L, albumin 2.9 g/dL, total bilirubin 1.9 mg/dL). The pronounced effect of argatroban was hypothesized to be due to poor cardiac function and therefore poor hepatic perfusion.

Gastrointestinal bleeding occurred while aPTTs were supratherapeutic, necessitating a 4-unit transfusion of packed red blood cells and the temporary cessation of argatroban. Upper and lower endoscopies showed only diffuse oozing likely due to underlying coagulopathy. Argatroban therapy was continued despite the gastrointestinal bleeding, in view of the patient's current manifestations of, and future risk for thromboembolism. That risk was underscored by ultrasound evidence of a free-floating pedunculated thrombus in the right internal jugular vein.

On the sixth day of argatroban therapy, the platelet count exceeded 100 × 10^9^/L. Warfarin was initiated at the expected maintenance dose (1 mg/day). After the first dose, however, her warfarin was held for two days because of INR values ranging between 2.5 and 6.0. All subsequent blood draws for PT/INR were done at least six hours after temporary cessation of argatroban infusion, and an INR goal between 2–3 was attained. Argatroban was discontinued after five days of overlap.

**Figure 2:**
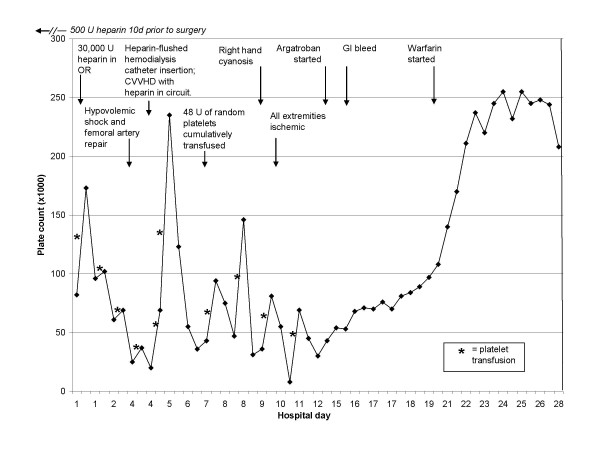
Platelet counts and key clinical events during hospitalization.

During the remainder of her 27-day stay in intensive care, the patient developed no additional thromboses. She required bilateral mid-foot amputations and amputation of all fingers of her right hand due to irreversible ischemic gangrenous necrosis. Figure 2 summarizes the patient's platelet counts and key clinical events during hospitalization. She was ultimately discharged to a long-term rehabilitation facility, but later died after cardiac arrest. No autopsy was obtained.

## Discussion

Thrombocytopenia, a commonly encountered condition in hospitalized patients, is often nothing more than a statistical laboratory variant or a benign hematologic state. The appropriate treatment may be simple observation with repeat measurement or withdrawal of an offending medication (e.g., a sulfonamide). However, in a significant number of cases, a low platelet count may herald a severe condition, e.g., HIT. HIT has traditionally been classified into two subtypes, based on pathophysiology. Type I is non-immune, usually of no clinical consequence, requires no treatment, and usually resolves spontaneously within days. Type II is an immune-mediated disorder with often catastrophic results. The cornerstones of its treatment are both discontinuation of all heparin (including catheter flushes) and initiation of alternative anticoagulation. Diagnosis is typically made on clinical grounds, with laboratory tests (often with slow turnaround times) playing a supportive role. Platelets usually decrease to either 50% of baseline or less than 150 × 10^9^/L. Assays for HIT include the sensitive (>90%) but less specific (~71%) heparin-platelet factor 4 ELISA which is often used as a screening test, and the serotonin release assay (sensitivity and specificity 100% and 97%, respectively) which can be performed as a confirmatory test but is not universally available and/or utilized [5]. The employment of a scoring system which estimates pretest probability may help guide clinical decision-making regarding the performance and/or interpretation of these diagnostic tests [6].

Without prompt diagnosis and proper treatment, patients with HIT complicated by thrombosis often experience dismal outcomes, with limb amputation in approximately 10%, and death in about 20%–30% [7]. The currently used classification scheme of two very different processes with significantly divergent treatments and outcomes has been partially responsible for the lack of awareness of HIT type II. For that reason, the term "HIT" when used alone, is now becoming reserved for use in referring to the immune-mediated type, or type II [4].

In HIT, even when thrombocytopenia is severe, bleeding is rare. Rather, patients with the greatest relative decrease in platelet count have the greatest risk of thrombosis [8]. Just as our patient was obviously prone to thromboembolism, so are approximately 50% of HIT patients, whose initial presentations include either venous or arterial thromboemboli [7]. Additionally, in HIT patients without thrombosis at diagnosis, the risk for thrombosis in the weeks after heparin cessation is 19%–52% [9-11]. This risk persists even after platelet counts have returned to normal, thus underscoring the need for long-term oral anticoagulation.

Our patient, who developed thrombocytopenia resistant to transfusion immediately after her open-heart surgery, highlights two interesting aspects of the diagnosis and presentation of HIT. First, there are often plausible alternative explanations in the critically ill patient with thrombocytopenia. In our patient, the decreased platelet count was initially attributed to both cardiopulmonary bypass and the presence of the intraaortic balloon pump. Later, it was blamed on sepsis, CVVHD, and bleeding. And though all of these devices and conditions are clearly associated with thrombocytopenia, HIT was not considered in the differential diagnosis despite the development of ischemic phenomenon. Secondly, while thrombocytopenia in HIT classically occurs within 5 to 10 days after initiation of heparin, our patient exemplifies the increasingly recognized rapid-onset presentation of HIT. This more fulminant type of HIT may occur in up to 30% of patients diagnosed with HIT, whereby the thrombocytopenia becomes apparent early, even within hours, after heparin re-exposure [11].

When HIT with or without thrombosis is suspected, the first step should be immediate cessation of all heparin, including heparin flushes and low-molecular-weight heparins. Alternative anticoagulation should be started immediately (Seventh ACCP Conference on Antithrombotic and Thrombolytic Therapy Grade 1C+ recommendation for lepirudin and Grade 1C recommendation for argatroban) [11]. Heparin cessation alone is insufficient, as patients remain in a prothrombotic state [9-11]. During the design of multicenter trials of direct thrombin inhibition in HIT, institutional review boards and the Food and Drug Administration deemed it unethical to have control arms consisting only of heparin cessation [12].

In the United States, the only approved anticoagulants for use in patients with HIT are the direct thrombin inhibitors argatroban, which is hepatically metabolized, lepirudin, which is renally cleared, and bivalirudin, which is only approved for patients undergoing percutaneous coronary intervention. Our patient was treated with argatroban, since lepirudin was contraindicated in the setting of acute renal failure.

This case report also illustrates an example of dosing eccentricities of argatroban. First, the initial starting dose of 2 mcg/kg/min resulted in aPTTs between 100–200 seconds despite normal values of liver enzymes. Four critically ill post-cardiac surgery patients with HIT have been previously described who became excessively anticoagulated with this same starting dose, despite normal hepatic enzymes [13]. The authors postulated that the drug's pharmacokinetics were altered by poor cardiac function and decreased hepatic perfusion, as did we. According to the manufacturer of argatroban, physicians should consider reducing the starting dose to 0.5–1 mcg/kg/min in critically ill patients who may have impaired hepatic perfusion (e.g., patients with vasopressors requirements, decreased cardiac output, volume overload, etc.). Indeed, our patient required an even lower infusion rate of 0.25 mcg/kg/min. Second, current treatment recommendations support starting oral anticoagulation once the platelet count rises above 100 × 10^9^/L [8]. However, after starting warfarin at the expected maintenance dose, we noticed higher than expected PT and INR values. Argatroban, typically monitored using aPTTs, also prolongs the PT and INR, thus confounding traditional interpretation of warfarin efficacy. INRs >5 commonly occur during argatroban therapy and argatroban-warfarin co-therapy in HIT, without bleeding complications, and guidelines for using INRs to monitoring the transition are available [14].

## Conclusion

Despite over 50 years of clinical experience with heparin, awareness of heparin-induced thrombocytopenia is still lacking. We present this case in an effort to solidify HIT's place in the differential diagnosis of thrombocytopenia. HIT was initially not considered despite documented prior heparin exposure and thrombocytopenia refractory to multiple platelet transfusions (i.e., suggesting an autoimmune or consumptive process). That the diagnosis still was not considered even when all digits were ischemic suggests a lack of awareness of HIT or an unwillingness of clinicians to challenge their initial diagnosis and objectively pursue alternative etiologies.

Without knowledge of HIT's subtleties, such as rapid-onset presentation and the ability to manifest as a result of seemingly insignificant amounts of heparin (e.g., catheter flushes), the correct diagnosis and treatment decisions may never be made. Without better awareness and understanding of its more dramatic and obvious presentations (Figure 1), the correct diagnosis may unfortunately be arrived at too late. Hence, our challenge remains to maintain a high index of suspicion for HIT, since the diagnosis can be made clinically and/or biologically in the setting of any patient with prior heparin exposure and a low or falling platelet count. In addition, effective treatment is available with the anticoagulants argatroban and lepirudin. Lastly, a readily available test with a high specificity is sorely needed.

## Competing interests

In the past five years neither author has received reimbursements, fees, funding, or salary from an organization that may in any way gain or lose financially from the publication of this manuscript, either now or in the future. No organization is financing this manuscript (including the article-processing charge). Neither author holds any stocks or shares in an organization that may in any way gain or lose financially from the publication of this manuscript, either now or in the future. Neither author holds or is currently applying for any patents relating to the content of the manuscript. Neither author has received reimbursements, fees, funding, or salary from an organization that holds or has applied for patents relating to the content of the manuscript. Neither author has any non-financial competing interests to disclose.

## Authors' contributions

LK cared for the patient in this case report. GC interviewed and examined the patient, and conducted a chart review. Both LK and GC drafted, read, and approved the final manuscript.
